# Idiopathic Intracranial Hypertension with Normal Cerebrospinal Fluid Pressure

**DOI:** 10.18502/jovr.v14i4.5475

**Published:** 2019-10-24

**Authors:** Masoud Aghsaei Fard

**Affiliations:** Department of Ophthalmology, Farabi Eye Hospital, Tehran University of Medical Sciences, Tehran, Iran

Sir,

I have read with enthusiasm the article published recently,^[1]^ based on which I would like to raise several concerns:

1. It is unclear how the diagnosis of idiopathic intracranial hypertension was made. In idiopathic intracranial hypertension, the opening pressure should be high. Based on this study, every patient with pale optic disc and normal lumbar puncture should be diagnosed as having idiopathic intracranial hypertension. The opening pressure of 18 cm H2O measured in this case report is not considered high. Additionally, the position (supine or lateral decubitus) during the lumbar puncture was not disclosed in the published article. In a large study conducted to find the reference range for cerebrospinal fluid pressure in 472 children,^[2]^ a threshold value of 28 cm H2O in the lateral recumbent position was set for high intracranial pressure. Authors recommended that for children an opening pressure above 28 cm H2O should be considered as elevated intracranial pressure. Another study also considered cerebrospinal fluid measures ≤ 28 cm H2O as "normal" for most children.^[3]^ The two case reports that the authors cited with normal cerebrospinal fluid pressure had other features of idiopathic intracranial hypertension such as papilledema, headache, and pulsatile tinnitus.^[4]^

2. The pale optic nerve thickness in this study was transformed confirming papilledema, which seems strange. In other words, the patient's retinal nerve fiber layer optical coherence tomography (RNFL OCT) values were 66 µm (in Figure 3(B), 41 µm) and 54 µm in the text; based on the authors' previous study, these were transformed to 400 and 560 µm thickness consistent with papilledema. No similar methods exist in the literature, except the authors' previous article in the *Journal of Contemporary Medicine Science*.^[5]^ If we accept this transformation in RNFL thickness, all optic atrophies with double hump appearance on RNFL OCT such as glaucoma and ischemic optic neuropathies would have papilledema.

3. Figure 1(C) had a very poor quality. The ganglion cell layer had artifacts and comparisons with Figures 3(B) and 3(C) (posttreatment) are not reasonable and do not support their claim that GC atrophy has improved.

4. Authors stated that asymptomatic mitochondrial mutation for Leber's hereditary optic neuropathy (LHON) could be present in general population, however, the patient in question had signs of optic neuropathy (i.e., optic atrophy).

5. Spontaneous visual recovery is possible in LHON with 14,484 mutation, and therefore visual improvement in this patient could also be explained with the natural course of LHON.

##  Financial Support and Sponsorship

Nil.

##  Conflicts of Interest

There are no conflicts of interest.
